# Multisystem Inflammatory Syndrome in an Adult Who Received Casirivimab-Imdevimab (REGEN-COV)

**DOI:** 10.7759/cureus.27353

**Published:** 2022-07-27

**Authors:** Gurdeep Singh, Alsayed Osman, Robert Ryad, Phuong Nguyen, Raphael Itzkowitz, Edward Maharam

**Affiliations:** 1 Internal Medicine, AdventHealth - Orlando, Orlando, USA

**Keywords:** atypical rash, immune therapy mediated myocarditis, systemic disease, mis-a, covid 19

## Abstract

Multisystem inflammatory syndrome in adults (MIS-A) is a systemic inflammatory syndrome that presents with a heterogeneous collection of signs and symptoms in adults. Here we present a case of a 38-year-old male who met the case definition of the MIS-A four weeks after a mild, symptomatic case of coronavirus disease 2019 (COVID-19) despite receiving casirivimab-imdevimab (REGEN-COV). Given the presence of signs and symptoms consistent with MIS-A, the patient was started on intravenous immune globulin (IVIG) and IV methylprednisolone. He promptly demonstrated clinical improvement over the next several days.

## Introduction

Shortly after the advent of the coronavirus 2019 (COVID-19) pandemic, several case reports of children and adults developing a “Kawasaki-like” illness in the weeks following an acute infection began to surface. Dubbed MIS, this clinical syndrome bears resemblance to Kawasaki’s disease in that there is a heterogeneous constellation of signs and symptoms secondary to systemic inflammation. In the months that followed, several case reports described a similar post-infectious syndrome in adults, known as a multisystem inflammatory syndrome in adults (MIS-A). 

The Centers for Disease Control and Prevention (CDC) has outlined clinical criteria for the diagnosis of MIS-A [1]. An individual should be greater than 21 years of age, hospitalized for greater than 24 h, and without a more likely alternative diagnosis for the illness [1]. Furthermore, primary clinical criteria for individuals suspected to have MIS-A necessitate a documented fever for greater than 24 h prior to hospitalization or within the first 3 days of hospitalization and either severe cardiac illness or rash and non-purulent conjunctivitis [1]. Secondary clinical criteria include new onset neurological symptoms, shock or hypotension, abdominal pain, vomiting, or diarrhea, and thrombocytopenia [1]. 

While there have been established case reports and case series on MIS-A, an extensive literature review has revealed very few cases of patients developing MIS-A despite receiving the casirivimab-imdevimab (REGEN-COV) infusion during the acute phase of COVID-19. Here we present a case of a 38-year-old male who met the case definition of MIS-A four weeks after a mild case of COVID-19 despite being treated with REGEN-COV. 

## Case presentation

A 38-year-old Caucasian male with a past medical history significant for mild, symptomatic COVID-19 treated with casirivimab-imdevimab (REGEN-COV) infusion four weeks prior to the current admission presented to Advent Health Orlando with painful left-sided neck swelling in November 2021.

Four weeks prior to being admitted to the hospital, he elected to get tested for SARS-CoV-2 after several family members tested positive and he began to develop non-specific upper respiratory symptoms including sinus congestion, rhinorrhea, and sore throat. He had no other comorbidities or reported allergies. The patient denied any history of receiving the COVID-19 vaccine. He qualified for the REGEN-COV infusion due to underlying obesity and received it a few days after testing positive for severe acute respiratory syndrome coronavirus 2 (SARS-CoV-2).

**Figure 1:**
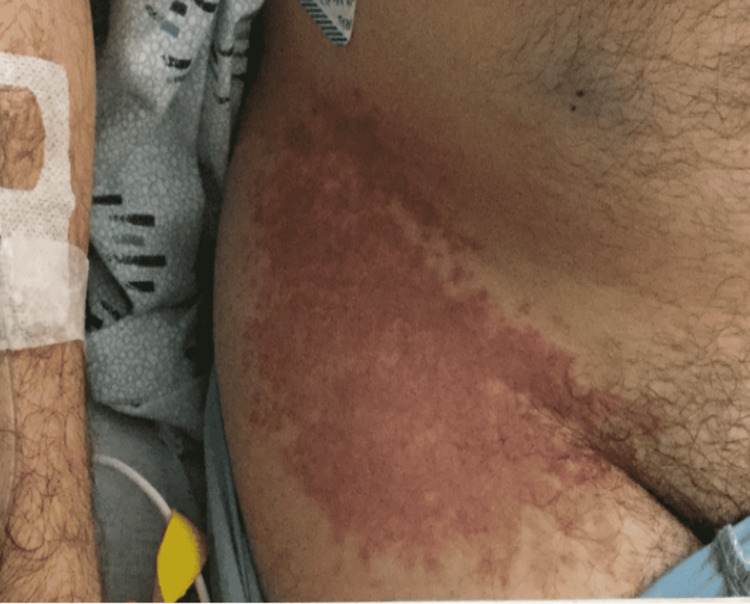
Rash on the patient's proximal right lower extremity.

During his initial evaluation in the hospital, the patient reported his left-sided neck swelling was associated with myalgias, nausea, diarrhea, asthenia, chills, a pruritic rash on the right lower extremity, and fever with a T-max of 104°F. Physical examination was pertinent for fever, tender left-sided anterior and posterior cervical lymphadenopathy, tachycardia, tachypnea, and a raised, palpable rash on the proximal aspect of the right lower extremity (Figure 1). Initial laboratory data were remarkable for leukocytosis with left shift and lymphopenia, mild acute kidney injury with creatinine of 1.3 mg/dL (reference range: 0.60-1.20 mg/dL), elevated erythrocyte sedimentation rate (ESR) at 123 mm/h (reference range: 0-15 mg/dL), and increased C-reactive protein (CRP) to 404.6 mg/L (reference range < 5 mg/dL). The patient also tested positive for SARS-CoV-2 RNA using polymerase chain reaction (PCR) testing and was found to have reactive antibodies against the SARS-CoV-2 nucleocapsid protein, suggesting attained immunity from his previous infection. Out of concern for sepsis, the patient was empirically started on IV piperacillin-tazobactam, azithromycin, and isotonic fluids. 

Despite the initiation of broad-spectrum antibiotics, the patient continued to have persistent fevers, diarrhea, new-onset headache with transient visual hallucinations, new-onset thrombocytopenia with a platelet count of 135,000/uL, and worsening pruritic rash that had spread to the distal left lower extremity by hospital day 2. His procalcitonin had acutely risen from 2.11 on hospital day 1 to 13.00 on hospital day 3. His ESR had also risen to above 130 mm/h and his interleukin-6 level was 552 pg/mL (reference range < 8 pg/mL).

**Figure 2:**
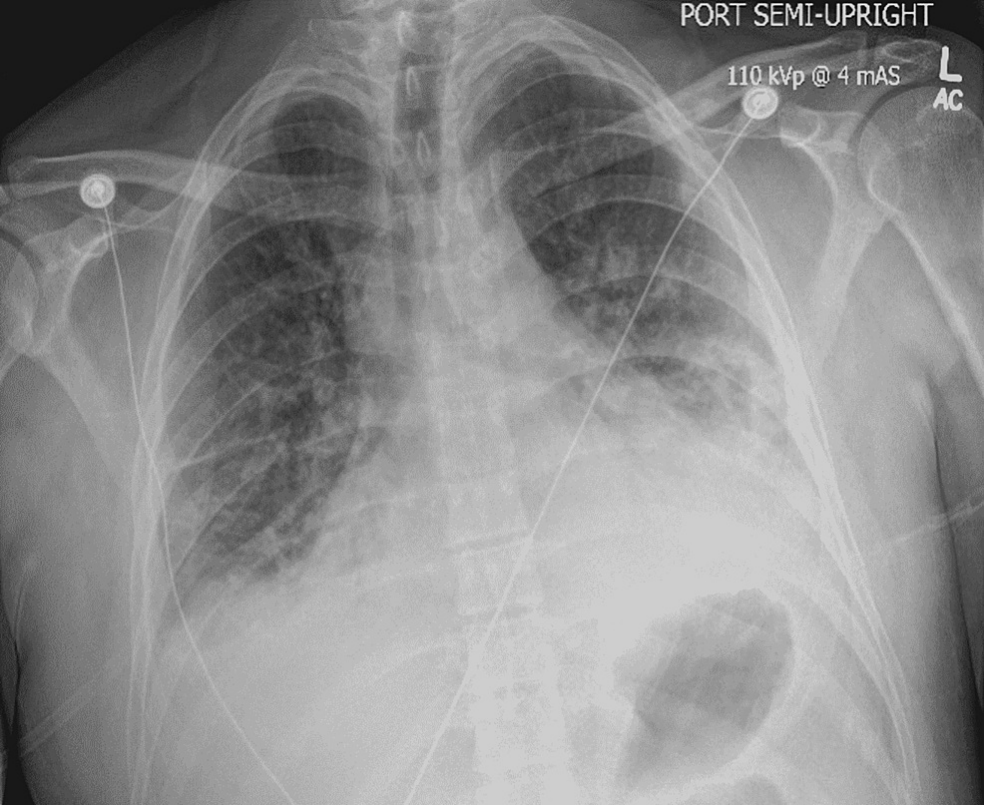
Chest X-ray demonstrating pulmonary edema and bilateral pleural effusions.

The patient's hospital course was also complicated by acute hypoxemic respiratory failure requiring four liters of supplemental oxygen via nasal cannula. Chest X-ray was obtained and revealed pulmonary edema most prominent in the bases with bilateral pleural effusions (Figure 2); N-terminal pro-BNP was elevated at 34,771 pg/mL (reference range: 0-450 pg/mL). Serial Troponin-T levels were positive at 0.09, 0.11, and 0.14 ng/mL, respectively (reference range: <0.03 ng/mL). Transthoracic echocardiography revealed mild global hypokinesis with reduced left ventricular systolic function at 45%. Cardiology was consulted, and it was determined that the patient’s cardiac manifestations were secondary to acute myocarditis. 

Given the patient’s rapid clinical deterioration, persistently worsening inflammatory indices, and the presence of signs consistent with the post-COVID MIS-A, Rheumatology and Infectious Disease was consulted. After a thorough literature review and collaboration with consulting physicians, the patient was started on intravenous immune globulin (IVIG) at 1 mg/kg/day administered over two days along with 1 g/day of IV methylprednisolone for three days for a presumed diagnosis of MIS-A. 

**Figure 3:**
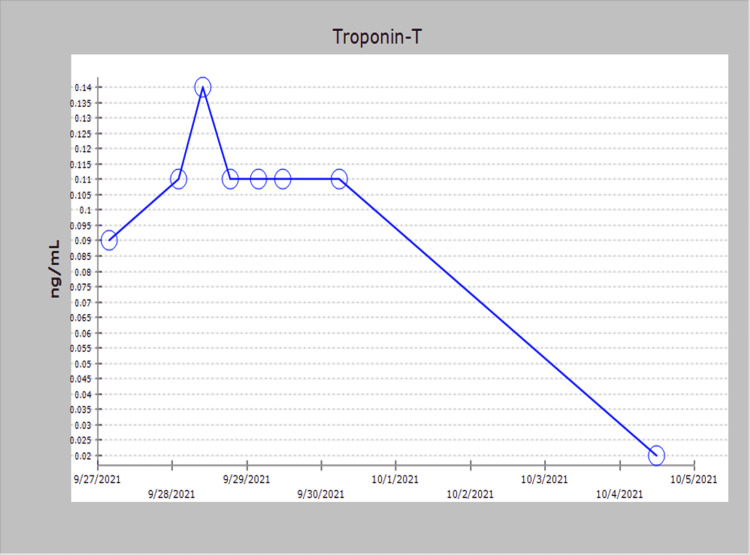
Troponin T level that normalized after starting IVIG and glucocorticoids. IVIG, intravenous immune globulin

**Figure 4:**
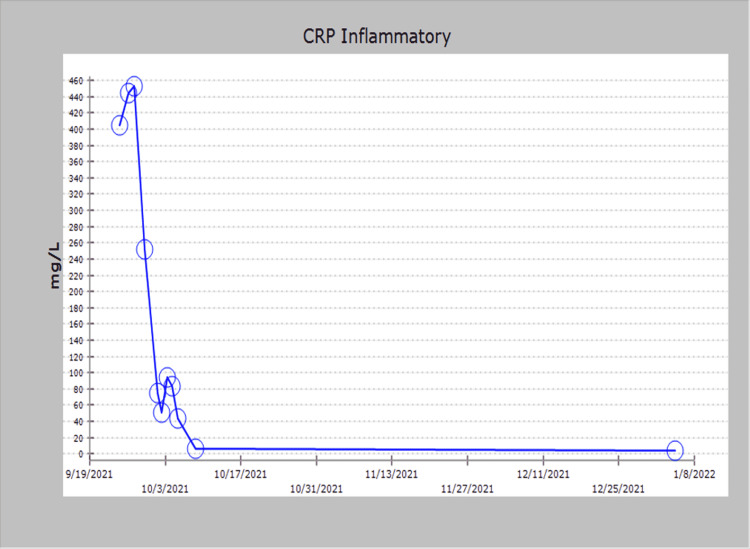
CRP level that normalized after starting IVIG and glucocorticoids. CRP, C-reactive protein; IVIG, intravenous immune globulin

The patient subsequently began to demonstrate clinical improvement over the next several days. His previous complaints of tender cervical lymphadenopathy, pruritic rash, fever, and diarrhea gradually resolved over a period of three days. Glucocorticoid therapy was changed to oral prednisone, repeat echocardiography revealed a restoration of left ventricular systolic function to an ejection fraction of 55%-60%, and he no longer required oxygen support. Serial chest X-rays revealed improving interstitial pulmonary edema, repeat Troponin-T levels were negative (Figure 3), and CRP had decreased to 5 ng/L (Figure 4).

The patient was subsequently discharged with a diagnosis of MIS-A and a prescription for oral prednisone 60 mg to be tapered over the course of six weeks. He was followed in the outpatient setting by primary care and Rheumatology. His CRP and ESR remained within normal limits after six weeks and he demonstrated no further sequela from either his COVID-19 infection or subsequent diagnosis of MIS-A. Glucocorticoids were discontinued and he is presently back to his baseline health. 

## Discussion

The growing number of documented cases of MIS-A prompted the CDC to establish a working case definition to assist healthcare providers in accurately diagnosing this rare, but life-threatening clinical entity [1]. Individuals should be over 21 years of age with an illness lasting at least 24 h in duration [1]. Furthermore, two of the following elements are required in conjunction with either severe cardiac dysfunction or a rash with concomitant non-purulent conjunctivitis: 1) abdominal pain or vomiting or diarrhea, 2) thrombocytopenia (platelet count < 150,000), 3) new-onset neurological signs and symptoms, and 4) presence of shock or hypotension [1]. Severe cardiac dysfunction was defined by the CDC to be either myocarditis, pericarditis, coronary artery dilation, new-onset right or left ventricular dysfunction characterized by an ejection fraction less than 50%, or arrhythmia. Supporting laboratory findings include elevations in any two of CRP, ferritin, interleukin-6, ESR, or procalcitonin as well as established evidence of recent COVID-19 infection with reverse transcriptase-PCR (RT-PCR), antigen testing, or serology [1].

This patient met the case definition of MIS-A as outlined by the CDC by virtue of having a prolonged fever, new-onset cardiac dysfunction with reduced ejection fraction, and findings consistent with myocarditis, new-onset thrombocytopenia, visual hallucinations, and persistent diarrhea. His markedly elevated ESR, CRP, interleukin-6, and acute rise in procalcitonin also supported the diagnosis. 

While their working case definition requires the absence of severe respiratory illness (to exclude the effects of tissue hypoxia as the cause of organ dysfunction), it is entirely plausible for patients to develop hypoxemia in the setting of heart failure mediated by cardiac inflammation. In Chau et al.’s case series of seven patients with a post-COVID hyperinflammatory syndrome leading to cardiogenic shock, they identified radiographic evidence of pulmonary infiltrates on four out of seven patients [2]. Furthermore, two out of seven required noninvasive ventilation [2]. 

The MIS-A presents with a heterogeneous collection of signs and symptoms. In Bastug et al.’s case review of 51 patients with MIS-A, cardiovascular abnormalities were found to be the most reported findings (42/51, 82.4%) [3]. Some 80.4% of patients had fever, 72.5% had gastrointestinal symptoms such as abdominal pain, nausea, vomiting, or diarrhea, 54.9% had respiratory symptoms, 39.2% had dermatological involvement, and 17.6% presented with lymphadenopathy [3]. The majority of the studied patients had laboratory evidence of elevated inflammatory markers, with a mean CRP value of 293.7 +/- 119.3 mg/L [3]. 

While there are no consensus treatment guidelines for MIS-A, the American College of Rheumatology has provided clinical guidance for the diagnosis and treatment of MIS-C [4]. IVIG at 2 mg/kg and IV methylprednisolone at 1-3 mg/kg/day remain first-line treatment options in the absence of contraindications [4]. For patients with refractory disease (defined as persistent fever or significant end-organ damage), 10-30 mg/kg/day of IV methylprednisolone, 4 mg/kg/day anakinra, or a one-time dose of 5-10 mg/kg IV infliximab may be considered [4]. As was the case with our reported patient, healthcare providers have seen robust responses after treatment options for MIS-C were extrapolated to adult populations [3, 5]. In their case report, Ahmad et al. successfully used 1 mg/kg of IVIG, 1 g/day of IV methylprednisolone, 250 mg/day of subcutaneous anakinra, and 325 mg/day of aspirin on a patient with MIS-A who developed cardiogenic shock and multi-organ dysfunction secondary to biventricular heart failure [5]. Ten days after starting the aforementioned therapies, his ejection fraction recovered from 15%-20% to 60% [5]. The use of anakinra was briefly considered in our reported patient but was ultimately deferred due to clinical improvement using IVIG and IV methylprednisolone alone. 

The pathophysiology of MIS-A remains poorly understood. Proposed mechanisms have included a dysregulated immune response after an antecedent COVID-19 infection, direct SARS-CoV-2 antibody-mediated processes, as well as persistent extrapulmonary infection [2]. In their prospective analysis of 10 children with MIS-C, Rostad et al. used quantitative enzyme-linked immunosorbent assays and found that all 10 children had markedly levels of immunoglobulin (IgG) antibodies against the SARS-CoV-2 receptor binding domain when compared to children with acute COVID-19, Kawasaki’s disease, and hospital controls [6]. Titers of IgG antibodies against the SARS-CoV-2 receptor binding domain were also found to be directly correlated with ESR and hospital length of stay, suggesting that the overt inflammation and clinical manifestations seen in MIS may be attributable to high neutralizing antibody titers [6]. 

Our case report is further notable for the development of MIS despite the receipt of monoclonal antibodies which were given when he first developed COVID-19 infection. Casirivimab and imdevimab were approved by the Food and Drug Administration for the treatment of mild-to-moderate COVID-19 in high-risk populations [7]. This antibody cocktail, known as REGEN-COV, was found in clinical trials to reduce the risk of hospitalization and death by COVID-19 [8]. This was also found to significantly reduce viral load compared to placebo [8]. In a phase 3 prevention trial, REGEN-COV was found to provide an 81% relative risk reduction in symptomatic infection by SARS-CoV-2 after 28 days [8]. Both casirivimab and imdevimab act by non-competitively binding to the spike protein found on SARS-CoV-2 at separate locations, leading to impaired binding between the spike protein and human angiotensin-converting enzyme-2 (ACE2) receptors [8]. The half-life of REGEN-COV has been estimated to be between 25.5 and 28.8 days, roughly the exact period between our patient receiving his infusion to the onset of MIS [8]. Further analysis into the use of REGEN-COV and the development of MIS-A is needed. 

## Conclusions

This case shows the importance of including MIS in the differential of an adult patient post-COVID-19 infection. While no treatment guidelines exist for MIS-A, multiple reports have demonstrated a response to high-dose steroids and IVIG. REGEN-COV has been found to reduce the risk of symptomatic COVID-19 but may not necessarily prevent MIS-A, as seen in this patient. 
